# Usnea Acid as Multidrug Resistance (MDR) Reversing Agent against Human Chronic Myelogenous Leukemia K562/ADR Cells via an ROS Dependent Apoptosis

**DOI:** 10.1155/2019/8727935

**Published:** 2019-02-11

**Authors:** Wenjing Wang, Shubin Niu, Luxin Qiao, Feili Wei, Jiming Yin, Shanshan Wang, Yabo Ouyang, Dexi Chen

**Affiliations:** ^1^Capital Medical University Affiliated Beijing You An Hospital, Beijing, 100069, China; ^2^Beijing Institute of Hepatology, Beijing, 100069, China; ^3^Beijing Precision Medicine and Transformation Engineering Technology Research Center of Hepatitis and Liver Cancer, 100069, China; ^4^School of biomedicine, Beijing City University, No. 6 Huanghoudian Road Haidian District, Beijing, 100094, China

## Abstract

**Purpose:**

Multidrug resistance (MDR) is a major obstacle in chemotherapy of leukemia treatments. In this paper, we investigated Usnea Acid (UA) as MDR reversal agent on hematologic K562/ADR cells via ROS dependent apoptosis.

**Methods:**

CCK8 assay was used to measure cell viability rate of K562/ADR. Intracellular reactive oxygen species (ROS) generation, cell cycle distribution, cell apoptosis were measured with flow cytometry, respectively. Proteins related to apoptosis were measured by Western blot. Intracellular Adriamycin accumulation was observed by confocal microscopy and measured by flow cytometry.

**Results:**

In vitro study showed intracellular Adriamycin accumulation was remarkably increased by UA. Cell viability treated with Adr (4 *μ*M) was decreased from 89.8%  ± 4.7 to 32%  ± 8.9 by combined with UA (4 *μ*M). Adr-induced apoptosis and G_1_/G_0_ phase cell cycle arrest were remarkably increased by UA, as well as, intracellular ROS level. However, MDR reversing activity of UA was inhibited by N-acetyl cysteine (NAC), a ROS scavenger.

**Conclusion:**

These data provide compelling evidence that UA is a promising agent against MDR in leukemia cell line and suggest a promising therapeutic approach for leukemia.

## 1. Introduction

Leukemia originates from abnormal hematopoietic stem cells which can result in a high number of deaths annually [1]. Over the past decades, major advances have been achieved in clinical treatment of leukemia. Despite overall improvement in the outcome of conventional leukemia chemotherapy, multiple drug resistance (MDR) is still the major problem in leukemia chemotherapy [2, 3].

Since first time being reported in 1970, MDR has been extensively studied by a multitude of academic researchers [4–6]. MDR is extremely complicated can be induced by different mechanisms. Overexpressing of ABC-transporters is recognized as the main cause of MDR, which is almost positive in all malignant tumor cells [7–9]. High level of ABC-transporters in leukemia cells may lead to increasing of drug efflux, decreasing intracellular drug concentration, thereby preventing the antiproliferation activity of chemotherapy drugs [10, 11]. Adriamycin (Adr) belongs to the anthracycline antibiotic family, displaying strong cytotoxicity and therefore generally being used as chemotherapeutic agents in clinical including leukemia [12]. However, expression of* MDR 1* mRNA or/and overexpression of proteins of ABS-transporter family induced MDR challenging Adr treatment against leukemia [13]. Based on this situation, developing of novel therapeutic strategies to reverse MDR is extremely important in the clinical of leukemia therapy.

Usnea Acid (UA), a bioactive lichen secondary metabolite, has been investigated as a promising anticancer agent in different cancer cell lines, including hepatocellular carcinoma, breast cancer, nonsmall cell lung cancer, and colon cancer [14].* In vitro* study using UA against malignant cells suggesting it can induce cell cycle arrest, autophagy, and apoptosis, thereby, has potential to be developed as a chemotherapeutic agent [15].

Reactive oxygen species (ROS) are a group of oxygen-containing, short-lived molecules that are highly reactive [16, 17]. Previous research has indicated that overproduction ROS can induce apoptosis* via* opening the mitochondrial permeability transition pore and thus releasing proapoptotic factors in leukemia cells [18, 19].

In this paper, we demonstrated that UA may increase the accumulation of Adriamycin in hematologic K562/ADR cells, reverse MDR via ROS dependent apoptosis induction.

## 2. Materials and Methods

### 2.1. Chemicals

Usnea Acid (UA), Adriamycin, and NAC were all purchased from sigma ((Sigma, St. Louis, MO, USA).

### 2.2. Cell Culture

Human cell lines (K562/ADR) were obtained from ATCC (Manassas, Virginia, USA) and cultured in Gibco™ RPMI-1640 complete medium (Thermo Fisher Scientific, HK, China) containing 10% heat inactivated FBS, 100 U/ml penicillin, and 100 mg/ml streptomycin. Before the study, K562/Adr cells were cultured in complete culture solution without Adriamycin for 48hr.

### 2.3. Adriamycin Accumulation

Adriamycin accumulation was measured by intensity of fluorescence of Adr. Cells were seeded into confocal dishes at a density of 5 × 10^5^ and then treated with UA (4 *μ*M), Adr (4 *μ*M), and UA plus Adr (4 *μ*M, respectively) for 48h; medium with same concentration of DMSO was used as control. After 3 washes with ice-cold PBS, cells were observed under a confocal microscopy (PerkinElmer UltraVIEW VOX, PE, Billerica, MA) and detected on BD FACSCalibur Cytometry.

### 2.4. CCK8 Cell Viability Assay

K562/Adr cells were cultured overnight after plated in triplicate wells in 96-well plates (4 × 10^3^ cells/well) followed by exposure to different concentrations of UA (0, 2, 4, 6, 8, 16, 32, and 64*μ*M), Adr (0, 2, 4, 6, 8, 16, 32, and 64*μ*M), and UA plus Adr (0, 2, 4, 6, 8, 16, 32, and 64*μ*M, respectively) for 48h. A total of 10 *μ*l CCK-8 reagents were added to the wells and kept in the incubator for 2-4 hr at 37°C after incubating. Finally, the absorbance was determined at 450 nm by a SpectraMax M5 Microplate Reader (Molecular Devices Instruments Inc., Sunnyvale, California, USA).

### 2.5. Cell Cycle Analysis

Cells were pretreated with UA (4*μ*M), Adr (4*μ*M), and UA plus Adr (4*μ*M, respectively) and incubated for 48h before being collected. Medium with same concentration of DMSO was used as control group. After incubation, cells were washed in PBS, and fixed in ice-cold 70% ethanol before being recentrifuged and incubated with RNase A (200 *μ*g/mL) and propidium iodide (PI, 5 *μ*g/mL). Cell cycle distribution was detected on BD FACSCalibur Cytometry. Data was analyzed with Cellquest software (BD Biosciences, Franklin Lakes, New Jersey, USA).

### 2.6. ROS Generation Measurement

Reactive Oxygen Species Assay Kit (KeyGEN BioTECH, Nanjing, China) was used to detect intracellular ROS levels. Exponentially growing cells were treated with UA (4*μ*M), Adr (4*μ*M), and UA plus Adr (4*μ*M, respectively) and incubated for 48h before harvesting and performed according to the manufacturer's instructions. The fluorescence of the cells was monitored using flow cytometry (FACSCalibur, BD, Franklin Lakes, New Jersey, USA). ROS production was calculated as the intensity in the fluorescence compared with the control group.

### 2.7. Flow Cytometric Analysis of Apoptosis

K562/ADR cells were seeded into 6-well plates at 5 × 10^5^ cells/well. After 12h incubation, cells were then treated with UA (4*μ*M), Adr (4*μ*M), and UA plus Adr (4*μ*M, respectively), followed by harvesting at 48h after treatment before being double-stained with Annexin V-FITC/PI (KeyGEN BioTECH, Nanjing, China) and subjected to flow cytometry analysis for detection of apoptosis. 10,000 cells per sample were analyzed by a BD FACSCalibur Cytometry (BD Biosciences, Franklin Lakes, New Jersey, USA) to quantify apoptotic cells (Annexin V-FITC positive cells).

### 2.8. Western Blot Analysis

K562/Adr cells were seeded into 6-well plates at 5 × 10^5^ cells/well. After overnight incubation, they were pretreated with 4 *μ*M Adr plus 4 *μ*M UA for 48h before being harvested and washed with ice-cold PBS and then lysed with ice-cold RIPA lysis buffer (KeyGEN BioTECH, Nanjing, China) with 1 mmol/L PMSF. Protein concentrations were calculated by BCA assay kits (Thermo Fisher SCIENTIFIC, Beijing, China). The western blot was performed as previously described [20]. Briefly, 20*μ*g of total protein was subjected to 12% SDS-PAGE gel and transferred to PVDF membranes (Millipore, Atlanta, Georgia, US). After blocking with 5% defatted milk for 1h, membranes were then incubated with primary antibody overnight at 4°C, followed by HRP-labeled secondary antibody at room temperature for 1h. Following each step, the membranes were washed five times with PBS-T for 5min. Immunoreactive proteins were detected using a chemiluminescence reagent by following the user manual; the GAPDH was selected as the loading control.

### 2.9. Statistical Analysis

All data are presented as mean ± standard deviation (SD) of three separate experiments. Data were evaluated using SPSS for Student's* t*-test and subjected to one-way or two way analysis of variance.

## 3. Results

### 3.1. Usnea Acid Effects on the Proliferation and Intracellular Accumulation of K562/ADR Cells

By combining confocal microscopy and flow cytometry analyses, we found that fluorescence intensity of Adr in K562/ADR cells became markedly higher in UA plus Adr group compared with Adr alone (1.75 folder,* p* < 0.01), indicating intracellular accumulation of Adr was increased by UA.

**Figure 1:**
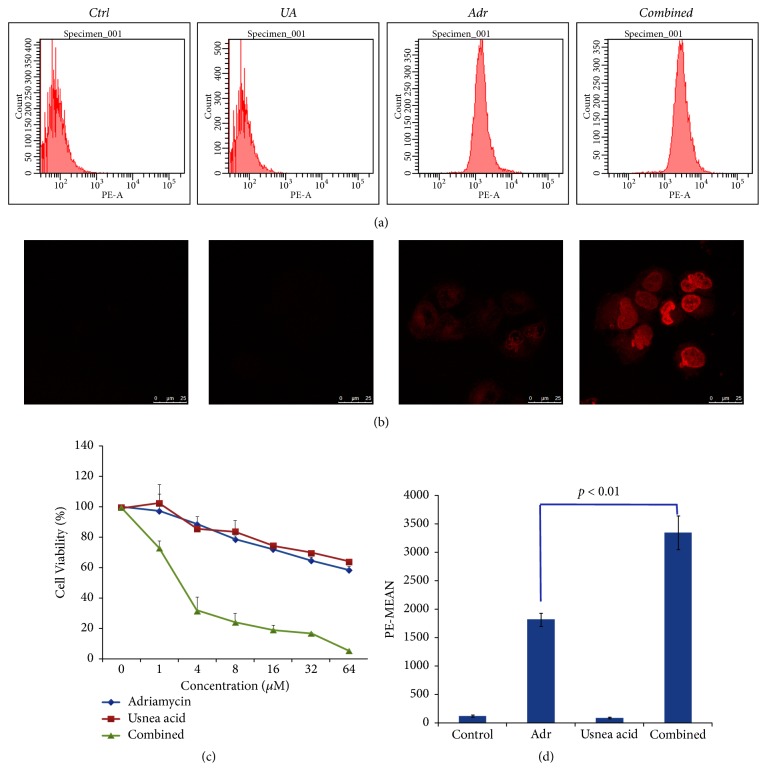
*Effects of UA on cell viability and intracellular accumulation of Adr in K562/ADR cells*. Cells were treated with UA(4 *μ*M), Adr UA(4 *μ*M), and UA plus Adr (4 *μ*M, respectively) for 48 hr, medium with same concentration of DMSO was used as control. Adr accumulation was increased by UR observed by confocal microscopy (b) and flow cytometry(a, d). Cell viability was dramatically decrease by UA plus Adr compare with Adr alone(c). Columns, values are expressed as mean ± SD.

The relative cell viability of treated cells was determined by CCK8 assay. As the results showed in Figure 1(c), cell viability was decreased by combination of UA and Adr compared with UA or Adr alone in a dose-dependent manner. According to the results of CCK8 assay, cell viability treated with Adr (4 *μ*M) was decreased from 89.8%  ± 4.7 to 32%  ± 8.9 by combined with UA (4 *μ*M). Base on that, we selected 4 *μ*M as the final concentration to do all the tests.

### 3.2. Usnea Acid Effects on the Generation of Intracellular ROS, Apoptosis Induction, and Cell Cycle Arresting of K562/ADR Cells

**Figure 2:**
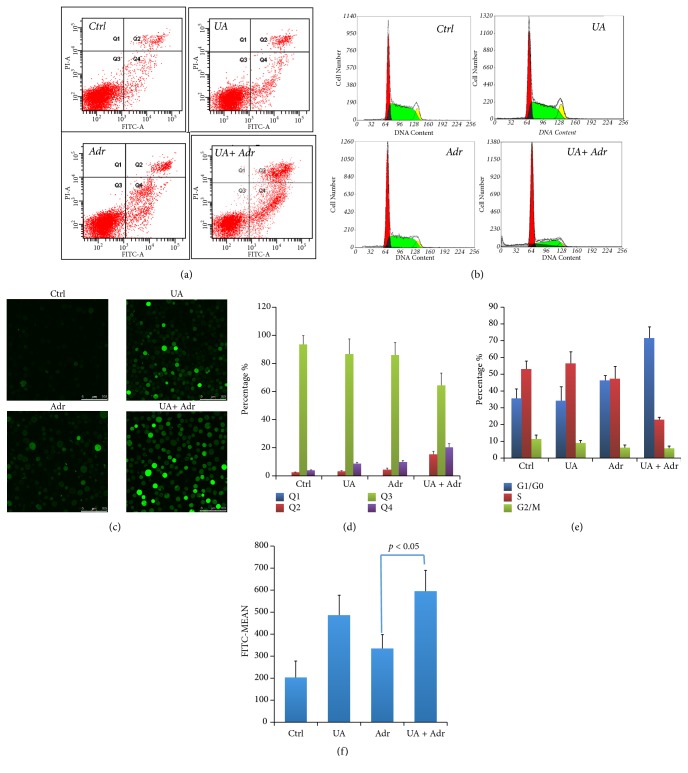
*UA Plus Adr Induced ROS Generation, Apoptosis, and Cell Cycle Arrest in K562/Adr Cells.* Cells were treated with UA(4 *μ*M), Adr UA(4 *μ*M), and UA plus Adr (4 *μ*M) for 48 hr before examination; medium with same concentration of DMSO was used as control. Annexin V-positive cells were analyzed by flow cytometry (a, d). Cell cycle arresting was observed by PI-staining DNA flow cytometric analysis (b, e). ROS generation was detected by confocal microscopy and flow cytometry (c, f). Columns: values are expressed as mean ± SD.

With the aim of determining the effective of apoptosis induction of UA combined with Adr, apoptotic cells percentage was evaluated by flow cytometric analysis using Annexin V/FITC-PI double staining assay. As shown in Figures 2(a) and 2(d), K56/ADR cells were resistant to Adr-induced apoptosis (9.7%); with combination of UA, the apoptotic cells were increased into 20.7%.

To explore the effect of UA combined with Adr on cell cycle distribution, propidium iodide DNA staining flow cytometric analyses were performed. As shown in Figures 2(b) and 2(e), UA plus Adr can induce cell cycle arrested in G_1_/G_0_ phase (71.5 %) compare with Adr treatment group (46.3%).

A great deal of research on multiple different cell lines has demonstrated that ROS can induce apoptosis [18, 19]. It was hypothesized that the primary mechanism of UA-mediated K56/ADR cells sensitization should be the induction of ROS dependent apoptosis. DCFH-DA assay was used to observe the content of ROS generation. As seen in Figures 2(c) and 2(d), the fluorescence intensity is 1.78-folder higher in UA combination group compared with Adr alone.

### 3.3. Increased Intracellular ROS Level Is Essential for Usnea Acid Reversing Adr Resistance in K562/ADR Cells

**Figure 3:**
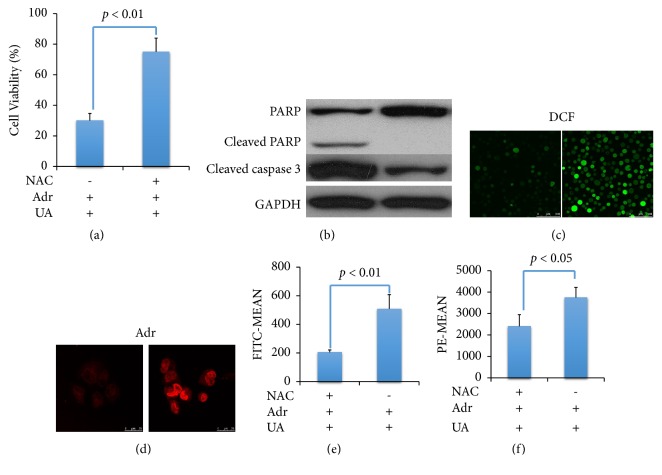
*MDR Reversing Activity of UA Was Inhibited by NAC.* Cells were treated with UA(4 *μ*M), Adr UA(4 *μ*M), and UA plus Adr (4 *μ*M, respectively) for 48 hr before examination; medium with same concentration of DMSO was used as control. Cell viability was detected by CCK8 assay (a). Apoptosis related proteins were analyzed by western blot (b). Intracellular generation of ROS (c) and accumulation of Adr (d) were observed by confocal microscopy and flow cytometry. Columns: values are expressed as mean ± SD.

In order to characterize the ROS generation is essential for UA reversing MDR in K562/ADR cells, NAC, a ROS scavenger was introduced. We used 10*μ*M DCFH-DA as a fluorescent probe to react with ROS and measured the intensity of the emitted light by confocal microscopy and flow cytometry. According to the results, ROS generation enhancement activity of UA was inhibited by NAC (Figure 3(c)); at the same time, the augmentation activity of UA on intracellular accumulation of Adr was reduced (Figure 3(d)).

By using CCK8 assay, we observed that cell viability was significantly increased when adding NAC in UA plus Adr group (Figure 3(a)), which indicated that enhanced cytotoxicity of Adr by UA was reversed by NAC. Furthermore, we detected protein expression of cleaved caspase 3 and PARP in K562/ADR cells. As shown in Figure 3(b), cleaved caspase 3 and PARP in cotreatment of UA and Adr group were decreased by NAC.

These results indicated that incubation with UA enhanced Adr induced apoptosis by regulation of intracellular ROS dependent apoptosis pathway.

## 4. Discussion

Currently, multidrug resistance (MDR) to antineoplastic drugs is a tough problem to successful treatment of leukemia. Although the mechanism of MDR has been extensively studied by a multitude of medical investigators, few drugs that can be used in clinical for reversing MDR were developed. Looking for novel agents with anti-MDR activity is therefore expected.

Usnea Acid (UA) is a multifunctional bioactive lichen secondary metabolite with potential anti-cancer properties.* In vitro *anticancer effects of Usnea Acid were shown for the first time by Kupchan and Kopperman against Lewis lung carcinoma [21]. Since then, many other researchers reported antiproliferative and mitochondrial depressive effects of UA against malignant cells* in vitro*, suggesting its potential use as a chemotherapeutic agent [22–24]. Although the promising therapeutic effects of UA have been investigated in different cancer cell lines, the multidrug resistance reversing activity in leukemia cells has yet to be elucidated. In this study, we investigated the MDR reversing activity of UA against human leukemia Adriamycin- (ADR-) selected multidrug resistance (MDR) cell line K562/ADR.

Most commonly encountered mechanism of multidrug resistance is characterized as intracellular drug depletion by efflux pump, leading to a cellular responsiveness. In our study, flow cytometry and confocal microscopy assay showed that intracellular accumulation of Adr was significantly increased by UA (Figures 1(a), 1(b), and 1(d)). Results from CCK8 assay indicated that UA can increase Adr antiproliferation activity against K562/ADR cells (Figure 1(c)).

Altered cell-cycle checkpoints and apoptosis resistance were also described as mechanisms of MDR [25, 26]. By using flow cytometry, we measured cell-cycle arresting and apoptosis inducing activity of Adr combined with UA compared with Adr alone. As results showed in Figures 2(a), 2(b), 2(d), and 2(e), cocultured with UA, cell-cycle arrested in G_1_/G_0_ phase by Adr was increase from 46.37% to 71.35%; at the same time, apoptotic cells induced by Adr increased from 9.7% to 20.3%. By combining confocal microscopy and flow cytometry, we found that ROS generation in K562/ADR cells was significantly increased by UA and Adr coculture (Figures 2(c) and 2(f)).

Reactive oxygen species (ROS) is a key stimulator in cell death. To obtain further information, we use NAC to inhibit ROS generation in K562/ADR cells. As results showed in Figure 3, ROS generation and Adr accumulation in K562/ADR cells increased by UA were inhibited by NAC analyzed by confocal microscopy and flow cytometry.

We also found that protein levels of cleaved caspase-3 and cleaved PARP expression in UA and Ader cocultured group were decreased by NAC; cell viability was increased at the same time (Figures 3(a) and 3(b)). These results indicated that incubation with UA enhanced ADR induced apoptosis by regulation of ROS generation in K562/ADR cells.

In conclusion, our data indicated that UA possessed the potential anti-MDR activity in K562/Adr cells through ROS-dependent apoptosis and G_1/_G_0_ phase cell-cycle arresting. UA might be a potential therapeutic compound for MDR leukemia treatment. However, there is a need for further studies investigating the molecular signaling mechanisms induced by UA treatment.

## Data Availability

The data used to support the findings of this study are included within the article.

## Supplementary Materials

Supplementary Materials Figure 1. Chemical structure of Usnea Acid and Adriamycin. Figure 2. Docking study of UA into ABCG 2 protein active domains. (A) Docking position of the binding site of ABCG 2, UA is shown as ball and stick mode in blue color. (B) The two-dimensional ligand-receptor interaction diagram of UA and human homology ABCG 2.
