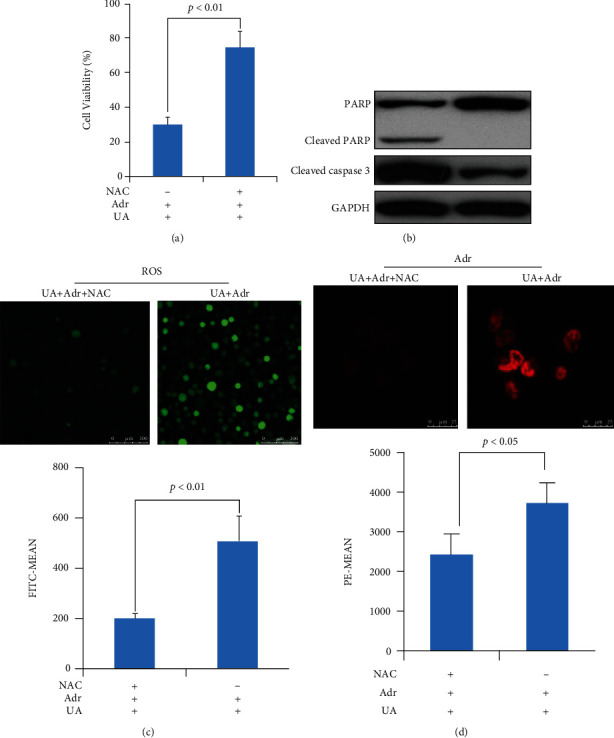# Corrigendum to “Usnea Acid as Multidrug Resistance (MDR) Reversing Agent against Human Chronic Myelogenous Leukemia K562/ADR Cells via an ROS Dependent Apoptosis”

**DOI:** 10.1155/2021/9808613

**Published:** 2021-12-22

**Authors:** Wenjing Wang, Shubin Niu, Luxin Qiao, Feili Wei, Jiming Yin, Shanshan Wang, Yabo Ouyang, Dexi Chen

**Affiliations:** ^1^Capital Medical University Affiliated Beijing You An Hospital, Beijing 100069, China; ^2^Beijing Institute of Hepatology, Beijing 100069, China; ^3^Beijing Precision Medicine and Transformation Engineering Technology Research Center of Hepatitis and Liver Cancer, 100069, China; ^4^School of Biomedicine, Beijing City University, No. 6 Huanghoudian Road Haidian District, Beijing 100094, China

The article titled “Usnea Acid as Multidrug Resistance (MDR) Reversing Agent against Human Chronic Myelogenous Leukemia K562/ADR Cells via an ROS Dependent Apoptosis” [1] contains a figure duplication in Figures 2 and 1, which was raised on PubPeer [2]. Specifically, Figure 2(c) panel UA + Adr overlaps with Figure 1(c) panel UA + Adr. The authors state that this was due to the incorrect file being selected during the preparation of the figure. The correct Figure 1(c) is as below.

## Figures and Tables

**Figure 1:**