# Supplementary material for: Usnea Acid as Multidrug Resistance (MDR) Reversing Agent against Human Chronic Myelogenous Leukemia K562/ADR Cells via an ROS Dependent Apoptosis
Source: Biomed Res Int. 2019 Feb 11;2019:8727935. doi: 10.1155/2019/8727935 (PMC6388510; doi:10.1155/2019/8727935)
Supplement: Supplementary Materials — Figure 1. Chemical structure of Usnea Acid and Adriamycin. Figure 2. Docking study of UA into ABCG 2 protein active domains. (A) Docking position of the binding site of ABCG 2, UA is shown as ball and stick mode in blue color. (B) The two-dimensional ligand-receptor interaction diagram of UA and human homology ABCG 2. [file 8727935.f1.docx]

**Supplementary Materials**

1. Structure of UA and Adriamycin


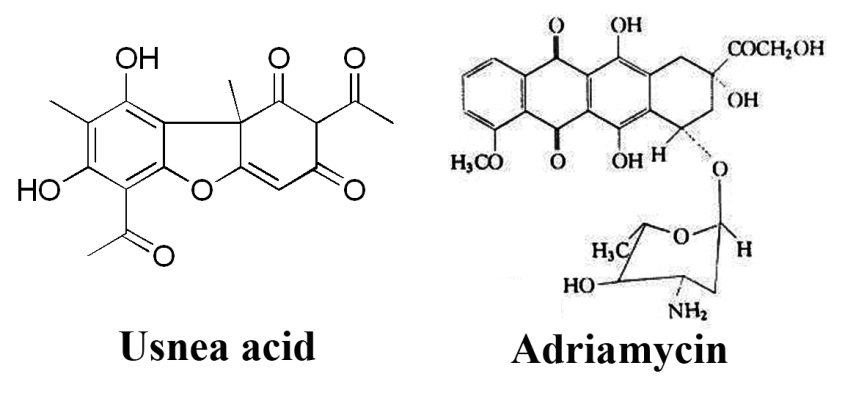


Fig 1. Chemical structure of Usnea acid and Adriamycin

1. Docking results of UA and Adriamycin in ABCG 2.

Docking studies suggesting that UA could bind into active domains of ABCG 2(Fig 2A). This interaction model is similar with original ligand Ko143 scaffold.

**
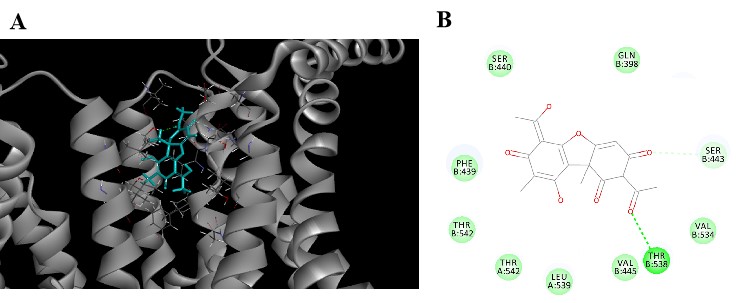
**

Fig 2. Docking study of UA into ABCG 2 protein active domains. A) Docking position of the binding site of ABCG 2, UA is shown as ball and stick mode in blue color. B) ) The two-dimensional ligand-receptor interaction diagram of UA and human homology ABCG 2.
